# Supplementary material for: Probing the Active Site of Class 3 L-Asparaginase by Mutagenesis: Mutations of the Ser-Lys Tandems of ReAV
Source: Biomolecules. 2025 Jun 29;15(7):944. doi: 10.3390/biom15070944 (PMC12292245; doi:10.3390/biom15070944)
Supplement: Supplementary file 1 [file biomolecules-15-00944-s001.zip › biomolecules-3656508-supplementary.pdf]

Probing the active site of Class 3 L-asparaginase by mutagenesis.

II. Mutations of the Ser-Lys tandems of ReAV

Kinga Pokrywka[1], Marta Grzechowiak[1], Joanna Sliwiak[1], Paulina Worsztynowicz[1,2], Joanna I. Loch[3], Milosz Ruszkowski[1], Miroslaw Gilski[1,4] and Mariusz Jaskolski[1,4*]

[1]Institute of Bioorganic Chemistry, Polish Academy of Sciences, Noskowskiego 12/14, 61-704 Poznan, Poland

[2]Department of Biotechnology and Food Microbiology, Poznan University of Life Science, Wojska Polskiego 48, 60-627 Poznan, Poland

[3]Department of Crystal Chemistry and Crystal Physics, Faculty of Chemistry, Jagiellonian University, Gronostajowa 2, 30-387 Krakow, Poland

[4]Department of Crystallography, Faculty of Chemistry, Adam Mickiewicz University, Uniwersytetu Poznanskiego 8, 61-614 Poznan, Poland

*corresponding author: mariuszj@amu.edu.pl

Keywords: hydrolase; amidohydrolase; L-asparaginase; leukemia; metalloprotein; site-directed mutagenesis; Nessler reaction; ITC; X-ray crystallography;

Supplementary Materials

Table S1. Specific activity of WT ReAV and its mutants measured using the Nessler reaction at 10 mM L-Asn. Enzymatic reactions were carried out for 10-30 minutes for the muteins and for 5 minutes for the WT protein [1].

Variant	Specific activity [μmol/s/mg]	Protein concentration used in assay [μM]
ReAV WT	6.5 ± 0.3	0.02
R47A	0.00034 ± 0.00011	10
S48A	0.00009 ± 0.00002	10
K51A	0.00008 ± 0.00006	15
S80A	-0.00038 ± 0.00045	7.1
K263A	-0.00023 ± 0.00006	10.4

Figure S1. Raw microcalorimetric kinetic data from shift-rate experiments (calorimetric multi-injection method, MIM) were obtained by titrating WT ReAV and its variants with either twelve 3 μL or twenty 1.8 μL aliquots of 100 mM L-Asn. Protein concentration used in the experiments is indicated above each plot. For the WT data, changes in differential power (DP) are shown, where the DP shift is directly proportional to the reaction rate. Particularly in the MIM approach, a final shift in baseline position indicates that the enzyme has reached its V_{max}. For the ReAV mutants, the ΔDP values were equal to or below the detection limit of the instrument of 0.1 μcal/sec, indicating that the studied muteins are at least 50 000 times less active than the WT protein.

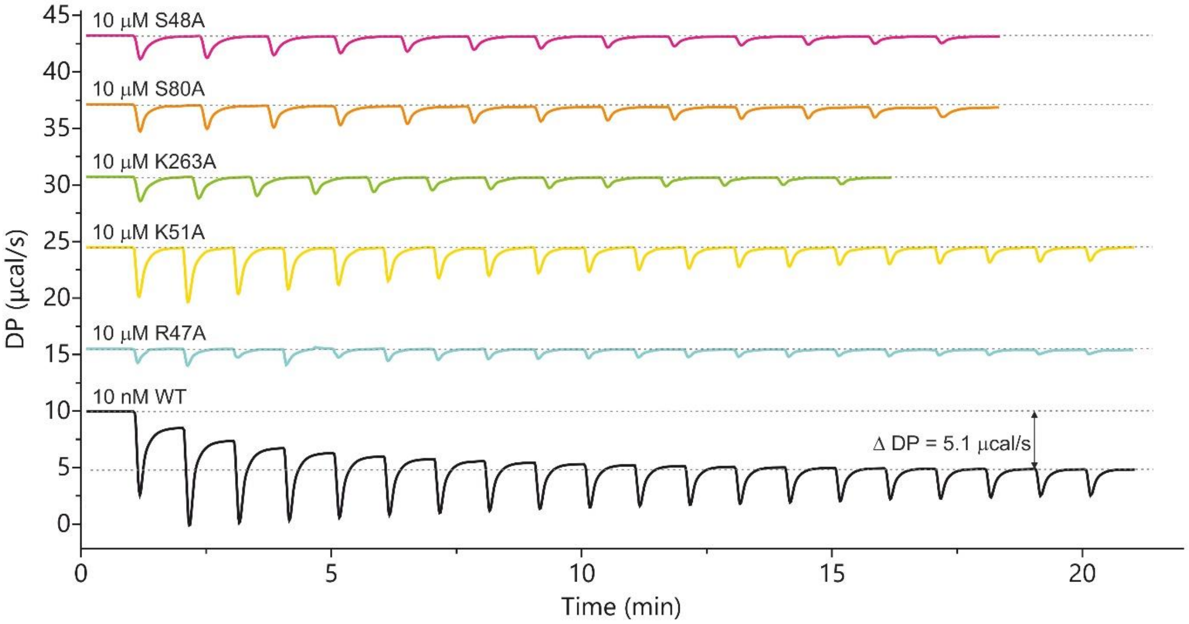

Reference

1. Sliwiak, J., Worsztynowicz, P., Pokrywka, K., Loch, J. I., Grzechowiak, M. & Jaskolski, M. Biochemical characterization of L-asparaginase isoforms from Rhizobium etli-the boosting effect of zinc. *Front Chem* **2024**, 12, 1373312.
