# Supplementary material for: Probing the Active Site of Class 3 L-Asparaginase by Mutagenesis: Mutations of the Ser-Lys Tandems of ReAV
Source: Biomolecules. 2025 Jun 29;15(7):944. doi: 10.3390/biom15070944 (PMC12292245; doi:10.3390/biom15070944)
Supplement: Supplementary file 1 [file biomolecules-15-00944-s001.zip › PDB report/D_1292141558_val-report-full_K51A-9QCW.pdf]

Full wwPDB X-ray Structure Validation Report (i)

Mar 6, 2025 – 10:42 am GMT

PDB ID	:	9QCW
Title	:	Crystal structure of Rhizobium etli L-asparaginase ReAV K51A mutant
Deposited on	:	2025-03-05
Resolution	:	1.95 Å(reported)

This wwPDB validation report is for manuscript review

This is a Full wwPDB X-ray Structure Validation Report.

This report is produced by the wwPDB biocuration pipeline after annotation of the structure.

We welcome your comments at *validation@mail.wwpdb.org*
A user guide is available at
https://www.wwpdb.org/validation/2017/XrayValidationReportHelp
with specific help available everywhere you see the (i) symbol.

The types of validation reports are described at
http://www.wwpdb.org/validation/2017/FAQs#types.

The following versions of software and data (see references (i)) were used in the production of this report:

MolProbity	:	4.02b-467
Mogul	:	1.8.4, CSD as541be (2020)
Xtriage (Phenix)	:	1.13
EDS	:	3.0
Percentile statistics	:	20231227.v01 (using entries in the PDB archive December 27th 2023)
CCP4	:	9.0.003 (Gargrove)
Density-Fitness	:	1.0.11
Ideal geometry (proteins)	:	Engh & Huber (2001)
Ideal geometry (DNA, RNA)	:	Parkinson et al. (1996)
Validation Pipeline (wwPDB-VP)	:	2.41

For Manuscript Review

1 Overall quality at a glance (i)

The following experimental techniques were used to determine the structure:
X-RAY DIFFRACTION

The reported resolution of this entry is 1.95 Å.

Percentile scores (ranging between 0-100) for global validation metrics of the entry are shown in the following graphic. The table shows the number of entries on which the scores are based.

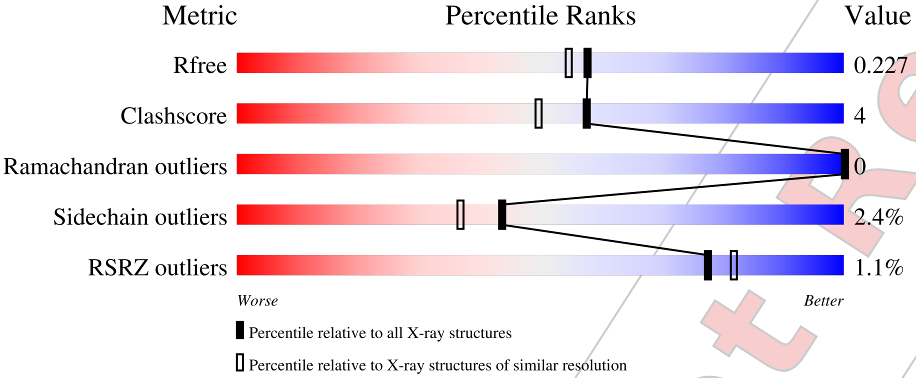

Metric	Whole archive (#Entries)	Similar resolution (#Entries, resolution range(Å))
R_{free}	164625	3187 (1.96-1.96)
Clashscore	180529	3412 (1.96-1.96)
Ramachandran outliers	177936	3390 (1.96-1.96)
Sidechain outliers	177891	3390 (1.96-1.96)
RSRZ outliers	164620	3186 (1.96-1.96)

The table below summarises the geometric issues observed across the polymeric chains and their fit to the electron density. The red, orange, yellow and green segments of the lower bar indicate the fraction of residues that contain outliers for >=3, 2, 1 and 0 types of geometric quality criteria respectively. A grey segment represents the fraction of residues that are not modelled. The numeric value for each fraction is indicated below the corresponding segment, with a dot representing fractions <=5% The upper red bar (where present) indicates the fraction of residues that have poor fit to the electron density. The numeric value is given above the bar.

Mol	Chain	Length	Quality of chain
1	A	373	% 85% 9% • 6%
1	B	373	2% 87% 10% • •

The following table lists non-polymeric compounds, carbohydrate monomers and non-standard residues in protein, DNA, RNA chains that are outliers for geometric or electron-density-fit criteria:

For Manuscript Review

Mol	Type	Chain	Res	Chirality	Geometry	Clashes	Electron density
2	EDO	B	403	-	-	X	-

For Manuscript Review

2 Entry composition (i)

There are 7 unique types of molecules in this entry. The entry contains 5946 atoms, of which 0 are hydrogens and 0 are deuteriums.

In the tables below, the ZeroOcc column contains the number of atoms modelled with zero occupancy, the AltConf column contains the number of residues with at least one atom in alternate conformation and the Trace column contains the number of residues modelled with at most 2 atoms.

- Molecule 1 is a protein called L-asparaginase II.

Mol	Chain	Residues	Atoms					ZeroOcc	AltConf	Trace
1	A	350	Total 2638	C 1632	N 480	O 505	S 21	0	9	0
1	B	366	Total 2745	C 1698	N 501	O 522	S 24	0	6	0

There are 16 discrepancies between the modelled and reference sequences:

Chain	Residue	Modelled	Actual	Comment	Reference
A	-5	GLY	-	expression tag	UNP Q9RFN5
A	-4	ILE	-	expression tag	UNP Q9RFN5
A	-3	ASP	-	expression tag	UNP Q9RFN5
A	-2	PRO	-	expression tag	UNP Q9RFN5
A	-1	PHE	-	expression tag	UNP Q9RFN5
A	0	THR	-	expression tag	UNP Q9RFN5
A	51	ALA	LYS	engineered mutation	UNP Q9RFN5
A	238	TYR	HIS	engineered mutation	UNP Q9RFN5
B	-5	GLY	-	expression tag	UNP Q9RFN5
B	-4	ILE	-	expression tag	UNP Q9RFN5
B	-3	ASP	-	expression tag	UNP Q9RFN5
B	-2	PRO	-	expression tag	UNP Q9RFN5
B	-1	PHE	-	expression tag	UNP Q9RFN5
B	0	THR	-	expression tag	UNP Q9RFN5
B	51	ALA	LYS	engineered mutation	UNP Q9RFN5
B	238	TYR	HIS	engineered mutation	UNP Q9RFN5

- Molecule 2 is 1,2-ETHANEDIOL (three-letter code: EDO) (formula: $C_2H_6O_2$).

For Manuscript Review

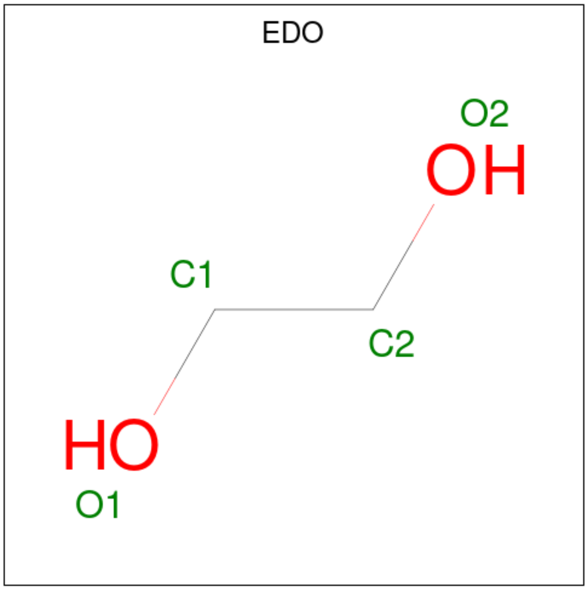

Mol	Chain	Residues	Atoms	ZeroOcc	AltConf
2	A	1	Total C O<br>4 2 2	0	0
2	A	1	Total C O<br>4 2 2	0	0
2	A	1	Total C O<br>4 2 2	0	0
2	A	1	Total C O<br>4 2 2	0	0
2	A	1	Total C O<br>4 2 2	0	0
2	A	1	Total C O<br>4 2 2	0	0
2	A	1	Total C O<br>4 2 2	0	0
2	B	1	Total C O<br>4 2 2	0	0
2	B	1	Total C O<br>4 2 2	0	0
2	B	1	Total C O<br>4 2 2	0	0
2	B	1	Total C O<br>4 2 2	0	0
2	B	1	Total C O<br>4 2 2	0	0
2	B	1	Total C O<br>4 2 2	0	0
2	B	1	Total C O<br>4 2 2	0	0

Continued on next page...

For Manuscript Review

Continued from previous page...

Mol	Chain	Residues	Atoms	ZeroOcc	AltConf
2	B	1	Total C O<br>4 2 2	0	0
2	B	1	Total C O<br>4 2 2	0	0
2	B	1	Total C O<br>4 2 2	0	0
2	B	1	Total C O<br>4 2 2	0	0

- Molecule 3 is SULFATE ION (three-letter code: SO4) (formula: O_4S).

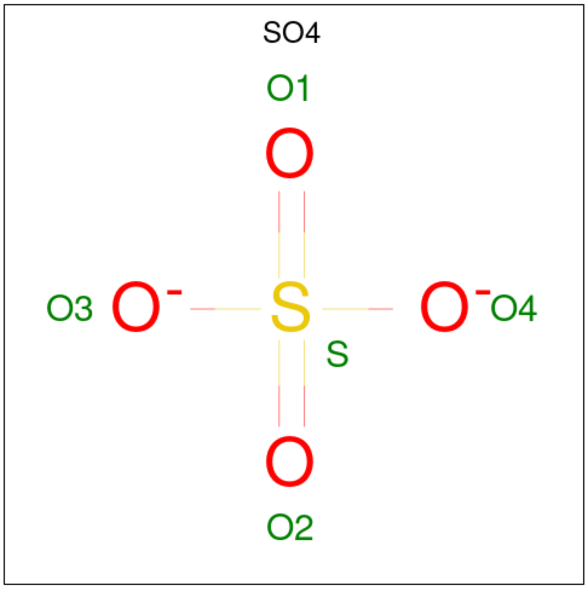

Mol	Chain	Residues	Atoms	ZeroOcc	AltConf
3	A	1	Total O S<br>5 4 1	0	1
3	B	1	Total O S<br>5 4 1	0	1

- Molecule 4 is GLYCEROL (three-letter code: GOL) (formula: $C_3H_8O_3$).

For Manuscript Review

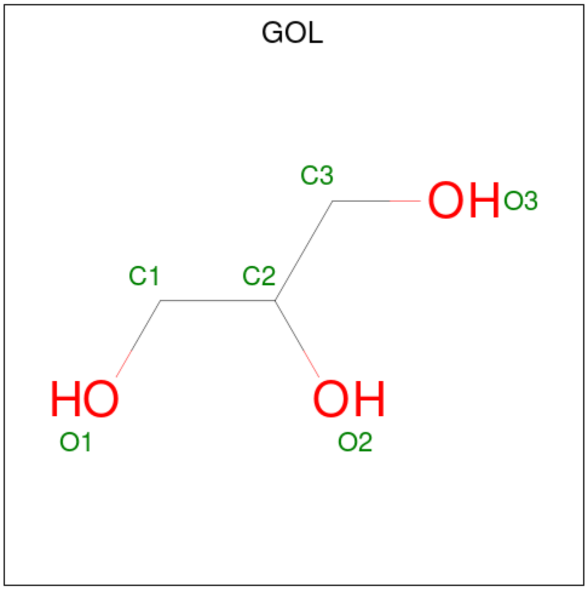

Mol	Chain	Residues	Atoms			ZeroOcc	AltConf
			Total	C	O		
4	A	1	6	3	3	0	0

- Molecule 5 is DI(HYDROXYETHYL)ETHER (three-letter code: PEG) (formula: $C_4H_{10}O_3$).

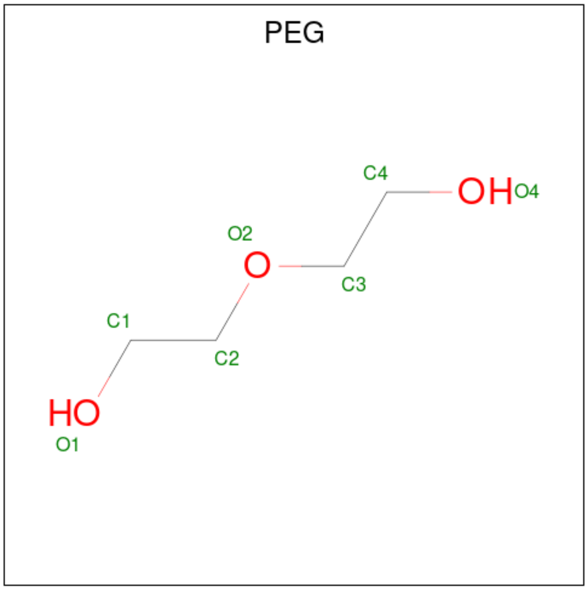

Mol	Chain	Residues	Atoms			ZeroOcc	AltConf
			Total	C	O		
5	A	1	7	4	3	0	0

- Molecule 6 is ZINC ION (three-letter code: ZN) (formula: Zn).

For Manuscript Review

Mol	Chain	Residues	Atoms	ZeroOcc	AltConf
6	A	1	Total Zn<br>1 1	0	0
6	B	1	Total Zn<br>1 1	0	0

- Molecule 7 is water.

Mol	Chain	Residues	Atoms	ZeroOcc	AltConf
7	A	202	Total O<br>202 202	0	0
7	B	264	Total O<br>264 264	0	0

For Manuscript Review

3 Residue-property plots (i)

These plots are drawn for all protein, RNA, DNA and oligosaccharide chains in the entry. The first graphic for a chain summarises the proportions of the various outlier classes displayed in the second graphic. The second graphic shows the sequence view annotated by issues in geometry and electron density. Residues are color-coded according to the number of geometric quality criteria for which they contain at least one outlier: green = 0, yellow = 1, orange = 2 and red = 3 or more. A red dot above a residue indicates a poor fit to the electron density (RSRZ > 2). Stretches of 2 or more consecutive residues without any outlier are shown as a green connector. Residues present in the sample, but not in the model, are shown in grey.

- Molecule 1: L-asparaginase II

Chain A:

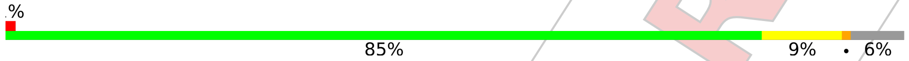

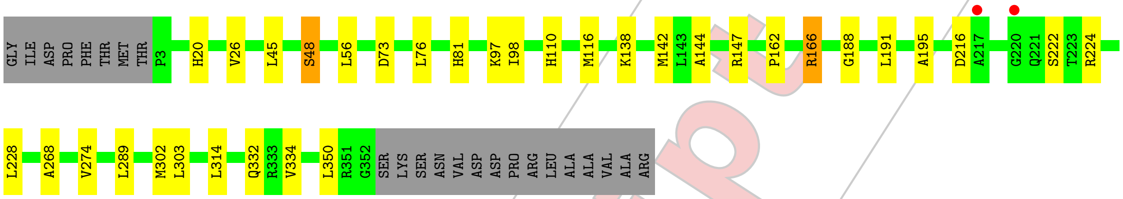

- Molecule 1: L-asparaginase II

Chain B:

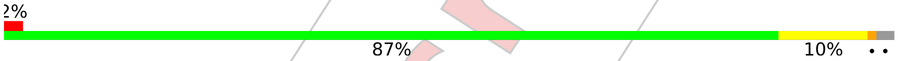

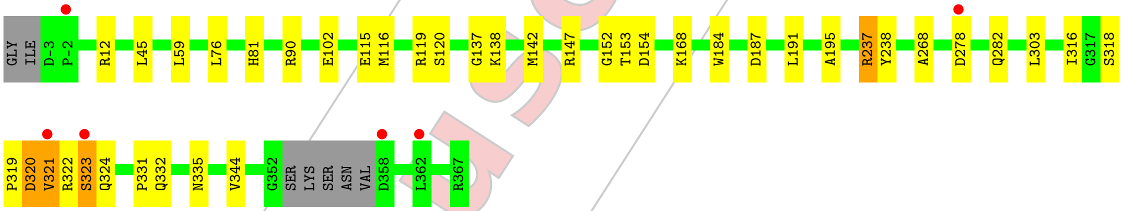

For Manuscript Review

4 Data and refinement statistics

Property	Value	Source
Space group	P 21 21 21	Depositor
Cell constants<br>a, b, c, α, β, γ	78.00Å 91.27Å 106.11Å<br>90.00° 90.00° 90.00°	Depositor
Resolution (Å)	69.20 – 1.95<br>69.20 – 1.95	Depositor<br>EDS
% Data completeness<br>(in resolution range)	99.5 (69.20-1.95)<br>99.7 (69.20-1.95)	Depositor<br>EDS
R_{merge}	0.14	Depositor
R_{sym}	(Not available)	Depositor
$< I/\sigma(I) >$ [1]	2.03 (at 1.95Å)	Xtriage
Refinement program	PHENIX 1.20.1_4487	Depositor
R, R_{free}	0.181 , 0.228<br>0.181 , 0.227	Depositor<br>DCC
R_{free} test set	1156 reflections (1.79%)	wwPDB-VP
Wilson B-factor (Å^2)	24.5	Xtriage
Anisotropy	0.763	Xtriage
Bulk solvent k_{sol}(e/Å^3), B_{sol}(Å^2)	0.32 , 38.7	EDS
L-test for twinning[2]	$< \|L\| > = 0.50$, $< L^2 > = 0.33$	Xtriage
Estimated twinning fraction	No twinning to report.	Xtriage
F_o,F_c correlation	0.96	EDS
Total number of atoms	5946	wwPDB-VP
Average B, all atoms (Å^2)	30.0	wwPDB-VP

Xtriage's analysis on translational NCS is as follows: *The analyses of the Patterson function reveals a significant off-origin peak that is 36.92 % of the origin peak, indicating pseudo-translational symmetry. The chance of finding a peak of this or larger height randomly in a structure without pseudo-translational symmetry is equal to 4.6392e-04. The detected translational NCS is most likely also responsible for the elevated intensity ratio.*

For Manuscript Review

[1] Intensities estimated from amplitudes.

[2] Theoretical values of $< |L| >$, $< L^2 >$ for acentric reflections are 0.5, 0.333 respectively for untwinned datasets, and 0.375, 0.2 for perfectly twinned datasets.

5 Model quality (i)

5.1 Standard geometry (i)

Bond lengths and bond angles in the following residue types are not validated in this section: PEG, GOL, ZN, EDO, SO4

The Z score for a bond length (or angle) is the number of standard deviations the observed value is removed from the expected value. A bond length (or angle) with $|Z| > 5$ is considered an outlier worth inspection. RMSZ is the root-mean-square of all Z scores of the bond lengths (or angles).

Mol	Chain	Bond lengths		Bond angles	
		RMSZ	#|Z| >5	RMSZ	#|Z| >5
1	A	0.49	0/2710	0.67	0/3661
1	B	0.57	0/2810	0.68	0/3796
All	All	0.53	0/5520	0.68	0/7457

There are no bond length outliers.

There are no bond angle outliers.

There are no chirality outliers.

There are no planarity outliers.

5.2 Too-close contacts (i)

In the following table, the Non-H and H(model) columns list the number of non-hydrogen atoms and hydrogen atoms in the chain respectively. The H(added) column lists the number of hydrogen atoms added and optimized by MolProbity. The Clashes column lists the number of clashes within the asymmetric unit, whereas Symm-Clashes lists symmetry-related clashes.

Mol	Chain	Non-H	H(model)	H(added)	Clashes	Symm-Clashes
1	A	2638	0	2615	19	0
1	B	2745	0	2722	29	0
2	A	28	0	42	2	0
2	B	44	0	66	8	0
3	A	5	0	0	0	0
3	B	5	0	0	1	0
4	A	6	0	8	1	0
5	A	7	0	10	1	0
6	A	1	0	0	0	0
6	B	1	0	0	0	0
7	A	202	0	0	1	0

Continued on next page...

For Manuscript Review

Continued from previous page...

Mol	Chain	Non-H	H(model)	H(added)	Clashes	Symm-Clashes
7	B	264	0	0	5	0
All	All	5946	0	5463	48	0

The all-atom clashscore is defined as the number of clashes found per 1000 atoms (including hydrogen atoms). The all-atom clashscore for this structure is 4.

All (48) close contacts within the same asymmetric unit are listed below, sorted by their clash magnitude.

Atom-1	Atom-2	Interatomic distance (Å)	Clash overlap (Å)
1:B:152:GLY:HA2	2:B:403:EDO:H11	1.58	0.86
1:B:45:LEU:HD23	1:B:195:ALA:HB2	1.62	0.81
1:A:45:LEU:HD23	1:A:195:ALA:HB2	1.68	0.75
1:B:90:ARG:HE	2:B:410:EDO:H12	1.56	0.70
1:B:153:THR:H	2:B:403:EDO:H22	1.58	0.68
1:A:116:MET:H	5:A:410:PEG:H31	1.59	0.67
1:A:224:ARG:HH11	4:A:409:GOL:H12	1.62	0.65
1:B:12:ARG:HH12	2:B:405:EDO:H11	1.66	0.59
1:B:153:THR:N	2:B:403:EDO:H22	2.17	0.58
1:B:316:ILE:O	1:B:322:ARG:HD3	2.03	0.58
1:A:56:LEU:HD22	1:A:228:LEU:HD21	1.87	0.56
1:B:268:ALA:HA	1:B:303:LEU:HD22	1.89	0.55
1:B:320:ASP:N	1:B:320:ASP:OD1	2.39	0.55
1:B:138:LYS:HE3	1:B:191:LEU:HD12	1.89	0.55
1:B:318:SER:H	1:B:321:VAL:HG13	1.72	0.54
1:B:76:LEU:HD11	1:B:81:HIS:CD2	2.45	0.52
1:A:76:LEU:HD11	1:A:81:HIS:CD2	2.44	0.52
1:B:90:ARG:NE	2:B:410:EDO:H12	2.21	0.51
1:A:188:GLY:HA2	1:B:335:ASN:HB2	1.93	0.51
1:A:162:PRO:O	1:A:166:ARG:HD2	2.10	0.50
1:A:48:SER:HB2	7:A:521:HOH:O	2.12	0.50
1:B:319:PRO:O	1:B:323:SER:HB3	2.11	0.49
1:A:110:HIS:CG	2:A:403:EDO:H21	2.48	0.49
1:B:59:LEU:HB3	2:B:412:EDO:H21	1.94	0.49
1:B:331:PRO:HD2	1:B:344:VAL:HG11	1.93	0.49
1:A:274:VAL:HB	1:A:289:LEU:HB3	1.94	0.49
1:A:268:ALA:HA	1:A:303:LEU:HD22	1.94	0.48
1:A:26:VAL:HG12	1:A:289:LEU:HD11	1.95	0.48
1:B:115:GLU:OE2	1:B:119:ARG:NH2	2.48	0.47
1:A:138[B]:LYS:NZ	1:A:191:LEU:HD12	2.30	0.47
1:B:102:GLU:HG3	7:B:664:HOH:O	2.15	0.47
2:A:408:EDO:H12	7:B:711:HOH:O	2.14	0.46

Continued on next page...

For Manuscript Review

Continued from previous page...

Atom-1	Atom-2	Interatomic distance (Å)	Clash overlap (Å)
1:A:20:HIS:CD2	1:A:302:MET:HG3	2.51	0.46
1:B:237:ARG:HG2	1:B:238:TYR:CE2	2.51	0.45
1:B:76:LEU:HG	1:B:137:GLY:HA3	1.98	0.45
1:B:282:GLN:HG3	7:B:710:HOH:O	2.17	0.45
1:A:73:ASP:OD2	1:A:97:LYS:NZ	2.47	0.44
1:A:332:GLN:HG3	1:A:334:VAL:HG23	1.99	0.43
1:A:98:ILE:HG21	1:A:144:ALA:HB2	2.00	0.42
1:B:138:LYS:O	1:B:142[A]:MET:HG2	2.18	0.42
1:A:314:LEU:HD21	1:A:350:LEU:HD21	2.01	0.42
1:B:237:ARG:HD2	7:B:622:HOH:O	2.19	0.41
1:B:332:GLN:HG2	7:B:671:HOH:O	2.21	0.41
1:B:321:VAL:O	1:B:324:GLN:HG3	2.22	0.40
1:B:168:LYS:HA	1:B:184:TRP:CZ2	2.57	0.40
1:B:154:ASP:H	2:B:403:EDO:H22	1.85	0.40
1:B:187[B]:ASP:HB2	3:B:409[B]:SO4:O2	2.21	0.40

There are no symmetry-related clashes.

5.3 Torsion angles (i)

5.3.1 Protein backbone (i)

In the following table, the Percentiles column shows the percent Ramachandran outliers of the chain as a percentile score with respect to all X-ray entries followed by that with respect to entries of similar resolution.

The Analysed column shows the number of residues for which the backbone conformation was analysed, and the total number of residues.

Mol	Chain	Analysed	Favoured	Allowed	Outliers	Percentiles	
1	A	357/373 (96%)	346 (97%)	11 (3%)	0	100	100
1	B	368/373 (99%)	360 (98%)	8 (2%)	0	100	100
All	All	725/746 (97%)	706 (97%)	19 (3%)	0	100	100

There are no Ramachandran outliers to report.

5.3.2 Protein sidechains (i)

In the following table, the Percentiles column shows the percent sidechain outliers of the chain as a percentile score with respect to all X-ray entries followed by that with respect to entries of similar

For Manuscript Review

resolution.

The Analysed column shows the number of residues for which the sidechain conformation was analysed, and the total number of residues.

Mol	Chain	Analysed	Rotameric	Outliers	Percentiles	
1	A	272/282 (96%)	266 (98%)	6 (2%)	47	41
1	B	282/282 (100%)	274 (97%)	8 (3%)	38	29
All	All	554/564 (98%)	540 (98%)	14 (2%)	44	34

All (14) residues with a non-rotameric sidechain are listed below:

Mol	Chain	Res	Type
1	A	48	SER
1	A	147	ARG
1	A	166	ARG
1	A	216[A]	ASP
1	A	216[B]	ASP
1	A	222	SER
1	B	116	MET
1	B	120	SER
1	B	147	ARG
1	B	237	ARG
1	B	278	ASP
1	B	320	ASP
1	B	321	VAL
1	B	323	SER

Sometimes sidechains can be flipped to improve hydrogen bonding and reduce clashes. There are no such sidechains identified.

5.3.3 RNA (i)

There are no RNA molecules in this entry.

5.4 Non-standard residues in protein, DNA, RNA chains (i)

There are no non-standard protein/DNA/RNA residues in this entry.

5.5 Carbohydrates (i)

There are no oligosaccharides in this entry.

For Manuscript Review

5.6 Ligand geometry (i)

Of 24 ligands modelled in this entry, 2 are monoatomic - leaving 22 for Mogul analysis.

In the following table, the Counts columns list the number of bonds (or angles) for which Mogul statistics could be retrieved, the number of bonds (or angles) that are observed in the model and the number of bonds (or angles) that are defined in the Chemical Component Dictionary. The Link column lists molecule types, if any, to which the group is linked. The Z score for a bond length (or angle) is the number of standard deviations the observed value is removed from the expected value. A bond length (or angle) with $|Z| > 2$ is considered an outlier worth inspection. RMSZ is the root-mean-square of all Z scores of the bond lengths (or angles).

Mol	Type	Chain	Res	Link	Bond lengths			Bond angles		
					Counts	RMSZ	$\#\|Z\|>2$	Counts	RMSZ	$\#\|Z\|>2$
2	EDO	A	408	-	3,3,3	0.42	0	2,2,2	0.68	0
2	EDO	B	405	-	3,3,3	0.66	0	2,2,2	0.64	0
2	EDO	B	412	-	3,3,3	0.58	0	2,2,2	0.26	0
4	GOL	A	409	-	5,5,5	0.70	0	5,5,5	1.32	0
2	EDO	B	403	-	3,3,3	0.61	0	2,2,2	0.27	0
3	SO4	B	409[B]	-	4,4,4	0.44	0	6,6,6	0.07	0
2	EDO	B	411	-	3,3,3	0.44	0	2,2,2	0.44	0
5	PEG	A	410	-	6,6,6	0.11	0	5,5,5	0.12	0
2	EDO	A	401	-	3,3,3	0.37	0	2,2,2	0.89	0
2	EDO	A	402	-	3,3,3	0.55	0	2,2,2	0.19	0
2	EDO	A	403	-	3,3,3	0.57	0	2,2,2	0.36	0
3	SO4	A	406[B]	-	4,4,4	0.44	0	6,6,6	0.07	0
2	EDO	B	401	-	3,3,3	0.50	0	2,2,2	0.79	0
2	EDO	B	410	-	3,3,3	0.37	0	2,2,2	0.37	0
2	EDO	B	404	-	3,3,3	0.69	0	2,2,2	0.30	0
2	EDO	A	405	-	3,3,3	0.46	0	2,2,2	0.97	0
2	EDO	B	402	-	3,3,3	0.53	0	2,2,2	0.13	0
2	EDO	A	407	-	3,3,3	0.65	0	2,2,2	0.48	0
2	EDO	B	408	-	3,3,3	0.45	0	2,2,2	0.93	0
2	EDO	B	407	-	3,3,3	0.38	0	2,2,2	0.96	0
2	EDO	A	404	-	3,3,3	0.84	0	2,2,2	0.83	0
2	EDO	B	406	-	3,3,3	0.49	0	2,2,2	0.92	0

In the following table, the Chirals column lists the number of chiral outliers, the number of chiral centers analysed, the number of these observed in the model and the number defined in the Chemical Component Dictionary. Similar counts are reported in the Torsion and Rings columns. '-' means no outliers of that kind were identified.

Mol	Type	Chain	Res	Link	Chirals	Torsions	Rings
2	EDO	A	408	-	-	0/1/1/1	-
2	EDO	B	405	-	-	0/1/1/1	-
2	EDO	B	412	-	-	0/1/1/1	-

Continued on next page...

For Manuscript Review

Continued from previous page...

Mol	Type	Chain	Res	Link	Chirals	Torsions	Rings
4	GOL	A	409	-	-	0/4/4/4	-
2	EDO	B	403	-	-	0/1/1/1	-
2	EDO	B	411	-	-	0/1/1/1	-
5	PEG	A	410	-	-	2/4/4/4	-
2	EDO	A	401	-	-	0/1/1/1	-
2	EDO	A	402	-	-	0/1/1/1	-
2	EDO	A	403	-	-	0/1/1/1	-
2	EDO	B	401	-	-	0/1/1/1	-
2	EDO	B	410	-	-	0/1/1/1	-
2	EDO	B	404	-	-	0/1/1/1	-
2	EDO	A	405	-	-	0/1/1/1	-
2	EDO	B	402	-	-	0/1/1/1	-
2	EDO	A	407	-	-	0/1/1/1	-
2	EDO	B	408	-	-	0/1/1/1	-
2	EDO	B	407	-	-	0/1/1/1	-
2	EDO	A	404	-	-	0/1/1/1	-
2	EDO	B	406	-	-	0/1/1/1	-

There are no bond length outliers.

There are no bond angle outliers.

There are no chirality outliers.

All (2) torsion outliers are listed below:

Mol	Chain	Res	Type	Atoms
5	A	410	PEG	O1-C1-C2-O2
5	A	410	PEG	C1-C2-O2-C3

There are no ring outliers.

9 monomers are involved in 13 short contacts:

Mol	Chain	Res	Type	Clashes	Symm-Clashes
2	A	408	EDO	1	0
2	B	405	EDO	1	0
2	B	412	EDO	1	0
4	A	409	GOL	1	0
2	B	403	EDO	4	0
3	B	409[B]	SO4	1	0
5	A	410	PEG	1	0
2	A	403	EDO	1	0
2	B	410	EDO	2	0

For Manuscript Review

5.7 Other polymers (i)

There are no such residues in this entry.

5.8 Polymer linkage issues (i)

There are no chain breaks in this entry.

For Manuscript Review

6 Fit of model and data (i)

6.1 Protein, DNA and RNA chains (i)

In the following table, the column labelled '#RSRZ> 2' contains the number (and percentage) of RSRZ outliers, followed by percent RSRZ outliers for the chain as percentile scores relative to all X-ray entries and entries of similar resolution. The OWAB column contains the minimum, median, 95th percentile and maximum values of the occupancy-weighted average B-factor per residue. The column labelled 'Q< 0.9' lists the number of (and percentage) of residues with an average occupancy less than 0.9.

Mol	Chain	Analysed	<RSRZ>	#RSRZ>2			OWAB($Å^2$)	Q<0.9
1	A	350/373 (93%)	-0.05	2 (0%)	85	88	15, 30, 45, 80	9 (2%)
1	B	366/373 (98%)	-0.12	6 (1%)	70	76	13, 26, 46, 70	6 (1%)
All	All	716/746 (95%)	-0.09	8 (1%)	77	82	13, 28, 46, 80	15 (2%)

All (8) RSRZ outliers are listed below:

Mol	Chain	Res	Type	RSRZ
1	A	217	ALA	3.7
1	B	321	VAL	3.5
1	B	362	LEU	2.9
1	A	220	GLY	2.5
1	B	358	ASP	2.3
1	B	278	ASP	2.1
1	B	-2	PRO	2.0
1	B	323	SER	2.0

6.2 Non-standard residues in protein, DNA, RNA chains (i)

There are no non-standard protein/DNA/RNA residues in this entry.

6.3 Carbohydrates (i)

There are no monosaccharides in this entry.

6.4 Ligands (i)

In the following table, the Atoms column lists the number of modelled atoms in the group and the number defined in the chemical component dictionary. The B-factors column lists the minimum,

For Manuscript Review

median, 95th percentile and maximum values of B factors of atoms in the group. The column labelled 'Q< 0.9' lists the number of atoms with occupancy less than 0.9.

Mol	Type	Chain	Res	Atoms	RSCC	RSR	B-factors(Å^2)	Q<0.9
2	EDO	A	401	4/4	0.71	0.14	46,47,47,55	0
5	PEG	A	410	7/7	0.71	0.15	36,42,45,45	0
3	SO4	A	406[B]	5/5	0.73	0.19	29,29,33,33	5
2	EDO	A	404	4/4	0.75	0.16	35,35,36,37	0
3	SO4	B	409[B]	5/5	0.76	0.17	24,26,33,35	5
4	GOL	A	409	6/6	0.79	0.13	40,45,49,53	0
2	EDO	A	403	4/4	0.80	0.14	36,39,41,44	0
2	EDO	A	408	4/4	0.80	0.17	37,37,39,42	0
2	EDO	B	401	4/4	0.80	0.15	35,36,36,40	0
2	EDO	B	403	4/4	0.80	0.13	45,48,49,50	0
2	EDO	B	405	4/4	0.81	0.14	34,39,39,42	0
2	EDO	B	408	4/4	0.83	0.17	40,41,45,48	0
2	EDO	B	411	4/4	0.83	0.15	40,42,42,44	0
2	EDO	B	402	4/4	0.83	0.17	34,40,43,46	0
2	EDO	A	405	4/4	0.84	0.13	41,45,48,50	0
2	EDO	B	404	4/4	0.87	0.15	34,35,36,40	0
2	EDO	A	402	4/4	0.87	0.14	41,42,45,46	0
2	EDO	B	406	4/4	0.88	0.11	37,39,41,44	0
2	EDO	B	412	4/4	0.88	0.13	34,36,39,43	0
2	EDO	A	407	4/4	0.88	0.14	27,33,35,36	0
2	EDO	B	407	4/4	0.92	0.11	23,27,30,39	0
2	EDO	B	410	4/4	0.93	0.15	28,29,32,39	0
6	ZN	B	413	1/1	0.96	0.06	33,33,33,33	1
6	ZN	A	411	1/1	0.98	0.04	37,37,37,37	1

6.5 Other polymers (i)

There are no such residues in this entry.

For Manuscript Review
